# Smart SDN Management of Fog Services to Optimize QoS and Energy

**DOI:** 10.3390/s21093105

**Published:** 2021-04-29

**Authors:** Piotr Fröhlich, Erol Gelenbe, Jerzy Fiołka, Jacek Chęciński, Mateusz Nowak, Zdzisław Filus

**Affiliations:** 1 Institute of Theoretical and Applied Informatics, Polish Academy of Sciences, 44-100 Gliwice, Poland; pfrohlich@iitis.pl (P.F.); mateusz@iitis.pl (M.N.); 2 Laboratoire I3S, Université Côte d’Azur, 06103 Nice, France; 3 Faculty of Automatic Control, Electronics and Computer Science, The Silesian University of Technology, Akademicka 2A, 44-100 Gliwice, Poland; jfiolka@polsl.pl (J.F.); jchecinski@polsl.pl (J.C.); zfilus@polsl.pl (Z.F.)

**Keywords:** Fog computing, software-defined networks (SDNs), green computing, energy-awareness, IoT, reinforcement learning, random neural networks, QoS optimization, artificial intelligence

## Abstract

The short latency required by IoT devices that need to access specific services have led to the development of Fog architectures that can serve as a useful intermediary between IoT systems and the Cloud. However, the massive numbers of IoT devices that are being deployed raise concerns about the power consumption of such systems as the number of IoT devices and Fog servers increase. Thus, in this paper, we describe a software-defined network (SDN)-based control scheme for client–server interaction that constantly measures ongoing client–server response times and estimates network power consumption, in order to select connection paths that minimize a composite goal function, including both QoS and power consumption. The approach using reinforcement learning with neural networks has been implemented in a test-bed and is detailed in this paper. Experiments are presented that show the effectiveness of our proposed system in the presence of a time-varying workload of client-to-service requests, resulting in a reduction of power consumption of approximately 15% for an average response time increase of under 2%.

## 1. Introduction

Fog computing is the decentralized computing structures which are located close to the IoT devices that generate data, and the Cloud servers that will ultimately store the data [1,2]. As such, it is a particularly useful paradigm for the Internet of Things (IoT) [3,4,5,6]. The Fog can support virtualized services for IoT clients, and effectively meet the wide variations of workload over time, offering the fast dynamic allocation of IoT and other clients to services.

The best way to balance load so as to offer low latency in such systems has long been considered in the context of distributed systems [7,8,9,10,11,12] and in autonomic communications [13] with regard to the Quality of Service (QoS). The allocation of tasks to different Cloud virtual machines, with the objective of minimizing response times, was considered in [14] using Reinforcement Learning (RL) [15] and deep learning [16]. Similarly, RL has been used to offer QoS in software-defined networks (SDNs) [17,18]. However, the increasing complexity of such systems raises issues about how several interacting optimization criteria, including QoS, power consumption [19] and security can be handled in a unified manner [20,21,22].

Fortunately, the increased availability of SDNs and their programmability offers a framework for implementing lightweight dynamic control schemes [23,24] that can combine the allocation of client requests to specific services located at certain servers, with the issue of establishing the connection between a specific client and a server through a network. SDN provides major advantages for the IoT, since the connection of IoT devices to Fog nodes can be handled by the SDN controller, since the open flow protocol [25] provides the required tool for routing, including the possibility of connecting each user or client request to a corresponding server. Furthermore, the centralization of control in the SDN architecture makes it easier to gather and efficiently utilize data regarding QoS and power usage.

Indeed, this is the basic idea addressed in this paper: since a client’s request must be connected to the service requested by the client, we used the source-to-destination routing capability of a SDN controller to create the connection which assigns the device where the client resides, to the server which hosts the service. However, in addition, we implemented a reinforcement learning-based “self-aware” technique within the SDN controller to make the best choice for the server where the service is located, so as to minimize a composite cost or goal function which aims to minimize both the response time of the request and the energy consumption in the SDN network.

### 1.1. Optimization Techniques for SDN Networks and the Fog

Fog computing has come to the forefront due to the need of moving data and services closer to the end users to attempt to provide low latency and fast access as compared to remote Cloud systems, in practical contexts such as mobile networks, the IoT and intelligent vehicle systems [6]. Many of the related research issues were discussed in a recent survey [26]. The basic techniques involved include the need to balance QoS, security and reliability in the resulting hardware–software architectures [27].

Specifically for SDN, various design and optimization choices have been discussed in a survey article [28], including issues of security and energy efficiency. The important issue of authenticating the routing choices made in SDN controllers is discussed in [21]. A survey of research that links system deployment issues and optimization was presented in [29].

In distributed systems and networks, the term “optimization” covers different approaches rather than a single view about how to address the issue. The traditional approach in distributed system optimization [8] assumes that the system workload is perfectly known in terms of deterministic quantities such as the average rates of request and average execution or transfer times, or the total data transfer rates, leading to a cost function and a non-linear minimization problem which is often combinatorially intractable and has to be solved approximately.

Such approaches are typically validated by a comparison with the global optimum in some cases, and via simulations in a variety of numerical examples, such as [30] where the SDN networking aspects (i.e., but not the Fog aspects) are taken into consideration, leading to a cost function including network delay and packet loss that helps find the best paths which minimize the cost function. Since the exact solution is of high complexity, it is typically replaced by a fast heuristic which is compared to the optimal to see whether it provides satisfactory results when all the deterministic parameters are known in advance. This approach was also developed in [31] for a case where failure probabilities are known, energy (or power) consumption at Fog nodes is small when they are idle, leading to a high complexity algorithm for exact optimization for packet loss and network forwarding delay including Fog node power consumption, which is then simplified to obtain a fast heuristic procedure.

This differs from our approach both in the optimization technique, which in our case uses online measurement, and in the cost function. In our present paper, the cost function includes the round-trip SDN network delay plus the service response time, and we also include the measured power consumption in the network nodes, which is not at all negligible when nodes are idle.

Another well-known approach to the optimization of networks and distributed systems is based on game theory [32]. Here, it is realistically assumed that the system parameters regarding users, such as traffic rates and workload parameters, are unknown and that different users will have different but unknown characteristics. The aim is then *not* to formulate or seek a global optimum, but to find the network’s stable operating where a change in strategy by an end user will not improve the user’s own outcome. Sometimes, this approach is combined with mechanisms such as auctions, which offer fast decision making when end users can express their needs in the form of a “bid”, and can have an economic connotation such as price, or price related to performance [33,34].

Optimization related to the selection of the parameters of a stochastic model, such as a queuing network, is yet another approach [35,36]. Although these models are stochastic and have the advantage of offering analytical solutions in *steady-state* when simple assumptions are made (such as Poisson arrivals and exponentially distributed service times), they require the system parameters to be known, such as arrival rates and service times [37]. When the system under consideration is optimized in real time, the resulting analysis is very difficult and difficult to exploit numerically even in simple cases [38,39].

However, another approach which we also follow is not to seek an *a priori* optimum, but rather to introduce an adaptation algorithm into the system which attempts to reduce the cost function at each step. A simple dynamic appoach to dealing with link failures in SDNs through this approach can be found in [40]. In this approach, a goal or cost function is defined, and online measurements are made so that at successive decision steps, network paths are changed so as to reduce the value of the cost function [41]. The tool often used for achieving this is reinforcement learning (RL) [15], which has also been applied to routing in intercontinental overlay networks [42], showing significant performance improvements over standard IP networks. As indicated earlier, another useful application of RL concerns packet routing in SDN networks [18,43].

### 1.2. Content of This Paper

SDN controllers working with RL were described in [17] with regard to the quality of service (QoS). The use of SDNs to optimize QoS and security was presented in [22].

**Figure 1:**
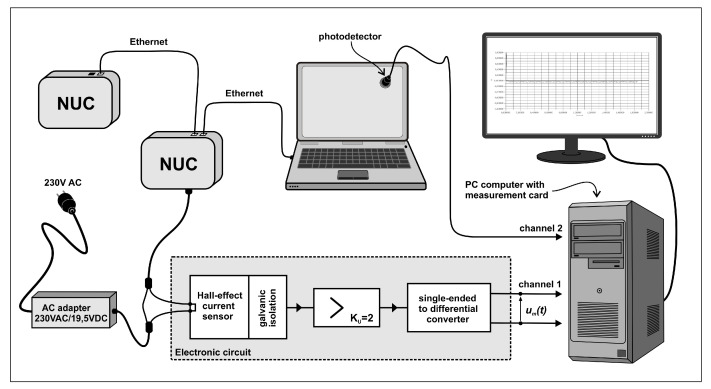
Measurement apparatus, based on the Hall effect, for power versus traffic characteristics of NUC hardware used for each SDN switch.

Our previous work that was presented at the Global Internet of Things Summit [44] focused on the QoS of Fog services, and we designed and evaluated a SDN-based controller for client–server task management to optimize the overall response time to service requests. While our earlier work was limited to improving the response time of users’ requests from servers, the present work addresses power consumption as well as response time minimization, expressed as a composite goal function. In addition, the text, figures and measurements reported in the present paper are totally disjoint from the earlier paper. In this paper:

1. We constructed a new objective or “goal function” for client-to-service allocation, which combines the total response time experienced and measured at the client end (including the round-trip network delay and the service time at the server), plus the power consumed in the SDN network by the client request.
2. Since load-dependent power measurements of the actual “NUC” hardware [45] that we use for each SDN switches is not available in the literature, we conducted accurate power versus traffic load measurements with a specific Hall effect apparatus. We note that the idle power consumption that we measured and report in Figure 1 is not negligible. Indeed while the NUC peak power consumption at maximum load is approximately 30 Watts, the idle consumption is approximately 20 Watts.
3. We detailed the adaptive control algorithm based on RL [15] and a random neural network [46,47,48] that acts as an adaptic critic, using the real-time measurement of the overall service response time, including the round-trip delay to send the request and receive the result through the SDN network as well as the server response time for processing the service request, and the traffic-driven power consumption in the SDN network. Note that others have used the RNN as a tool for controlling the online performance of packet networks and mobile networks [49,50,51,52,53,54].
4. This work extends on previous research that only addressed the network aspects with regard to QoS [41] and QoS and security [55]. We discuss in detail the computational complexity of the algorithm and show that it is $O(n^{2})$ where *n* is the number of different possible connection paths between clients and services.
5. The RL algorithm is implemented in the SDN controller, and takes online decisions in real time that minimize the composite goal function.
6. We show the effectiveness of our technique by exhibiting measurement results on a multi-hop SDN network, together with client software requests and servers, with multiple users and multiple servers. Our experiments show in particular that our adaptive controller achieves power savings of the order of 15% with a very moderate (but consistent) less than 2% increase in average response time.

Thus, in Section 2 we discuss how an instance of a service is selected to satisfy the request made by a user, when multiple instances of the available services are located at different nodes of a Fog platform, and each of the servers that house the services may be reached by one of several multi-hop paths in a SDN network. We also discuss its implementation using a random neural network with reinforcement learning.

Section 3 discusses the issue of accurately measuring power consumption in routers or switches, and describes the technique we used to acquire the measurement data. The resulting measurements on the specific SDN switch used in this work are also presented.

Section 4 presents our experimental setting and summarizes the measurement results concerning the RL-based control technique for allocating services to clients in the Fog, using the SDN controller to set up the connections, while optimizing the goal function that combines the response time and power consumption. Finally, Section 5 summarizes our results and suggests directions for future work.

## 2. The Decision System

The system considered is composed of a set *F* of Fog servers, where each server f∈F={1,...|F|} supports any software service i∈S. We assume that each service is available for execution at all of the Fog servers, but the extension to the case where each Fog server only handles some of the services is straightforward.

We also have a set of users or clients *U*, where the *u* denotes a client u∈U={1,...,|U|}. Clients will be located at different devices in the system, and we can imagine (u,f) to be a pair of IP addresses. A client *u* requesting service *i* can be connected to a Fog server *f* by some multi-hop path π(u,i,f) that originates at *u* and ends at *f*. We note that even for a fixed Fog server *f*, there can be several distinct paths from *u* to *f*.

However, because the service *i* may be located at any Fog node, the set of paths that *u* can use to reach *i* is in fact the set of all paths from *u* to all the servers *F*, which we denote by Π(u), and N(u)=|Π(u)| is the total number of paths connecting *u* to all the Fog servers.

When the service *i* located at Fog server *f* is used to satisfy the request, we denote by π(u,i,f)∈Π(u) the path used to transfer the request from *u* to service *i*. We assume that the same path in reverse will be used to transfer the result back to the client *u*. Note that the path is physically composed of a sequence of SDN switches, since we deal with a SDN network.

In general, π(u,i,f) will not be selected “at random”, but rather it will be selected based on the resulting QoS and network power consumption, hence:

- For each Fog node and client, we need to estimate the response time $T_{1}\left(u,i,f\right)$ which is the overall time (including any waiting time) it takes the server *f* to service *i* for client *u*.
- Similarly, for the specific path π(u,i,f), we will need to estimate the round-trip transfer time $T_{2}\left(\pi \left(u,i,f\right)\right)$ for transferring the service request and any needed data from *u* to the service *i* at *f*, and for transferring the results back from *f* to *u*.
- Finally, we will also require an estimate of the *network power consumption* E(π(u,i,f)) for the request of client *u* for service *i*, which includes the round-trip transfer associated with the amount of data D(u,i) involved in the request, and the energy consumption characteristics of the SDN switches on the path π(u,i,f).

Note that $T_{1}\left(u,i,f\right)$ does not depend on the path π(u,i,f); rather, it depends on the request, the service, and the Fog node where the service is executed, i.e., (u,i,f). On the other hand, $T_{2}\left(\pi \left(u,i,f\right)\right)$ depends on the path. Both can be estimated from past measurements, as we performed in the RL-based control scheme described in Section 2.1. From the above quantities, we can express the overall cost or goal function G(u,i,f):

(1) $$G\left(u,i,f\right)=\alpha [\;T_{1}\left(u,i,f\right)+T_{2}\left(\pi \left(u,i,f\right)\right)\;]+\left(1-\alpha \right)100.E\left(\pi \left(u,i,f\right)\right),$$

where 0≤α≤1 is a constant that weighs the relative importance of the total delay and power consumption within the overall cost, and the factor 100 is used to match the power value in Atts with the delay metric in milliseconds.

For client *u* requesting service *i*, the optimum path $\pi^{*}\left(u,i\right)$ which depends on *u* and *i* that will be used is then determined as follows:

(2) $$\pi^{*}\left(u,i\right)=\arg\;\min\left\{G\left(u,i,f\right)\;:\;\forall \pi \left(u,i,f\right)\in \Pi \left(u\right)\right\}\;.$$

Notice that the choice of the optimum path also determines the choice of the corresponding Fog server *f* in a unique manner. Thus, we reduced the **problem of selecting a Fog server to allocate a user’s request** to:

- Selecting the **optimum path in the network** to connect a user to a specific Fog server for a given service, since the choice of the path determines the choice of the Fog server that is selected.
- Moreover, the practical consequence is that this can be implemented by a SDN controller whose the normal function was to select a path in the network for a given connection.

### 2.1. Random Neural Network and Reinforcement Learning

The G(.) function (1) is learned or estimated by collecting measurements, and an approximation for the optimum path (2) is selected through RL [15] using a distinct random neural network (RNN) [46,56] that acts as an adaptive critic for each client–service pair (u,i).

Each RNN (u,i) has as many neurons as there are paths from *u* to all the Fog servers, i.e., N(u)=|Π(u)| neurons, so that the number does not depend on the service, but just on the client *u*. Therefore, each neuron of RNN (u,i) has a state $q_{\pi }\left(u,i\right)$ for each path π∈Π(u), which is the probability that the particular neuron is excited. The states satisfy the standard RNN system of equations:

(3) $$q_{\pi }\left(u,i\right)=\frac{\Lambda_{\pi }\left(u,i\right)+\sum_{\pi^{\prime }\in \Pi \left(u\right)}q_{\pi^{\prime }}\left(u,i\right)W_{\pi^{\prime },\pi }^{+}\left(u,i\right)}{\lambda_{\pi }\left(u,i\right)+r_{\pi }\left(u,i\right)+\sum_{\pi^{\prime }\in \Pi \left(u\right)}q_{\pi^{\prime }}\left(u,i\right)W_{\pi^{\prime },\pi }^{-}\left(u,i\right)},$$

where:

(4) $$\begin{array}{ccc} & & W_{\pi ,\pi }^{+}\left(u,i\right)=W_{\pi ,\pi }^{-}\left(u,i\right)=0,\;\forall \pi \in \Pi \left(u\right),\;and \end{array}$$

(5) $$\begin{array}{ccc} & & r_{\pi }\left(u,i\right)=\sum_{\pi^{\prime }\in \Pi \left(u\right)}\left[W_{\pi ,\pi^{\prime }}^{+}\left(u,i\right)+W_{\pi ,\pi^{\prime }}^{-}\left(u,i\right)\right],\;is\;the\;firing\;rate\;of\;neuron\;\pi \;. \end{array}$$

Equation (3) states that one RNN is associated with each client service pair (u,i), and this RNN has one neuron associated with each distinct network path π that connects *u* to *i*. Note that a path π may be used by several client–service pairs. The quantities $W_{\pi^{\prime },\pi }^{+}\left(u,i\right)$ and $W_{\pi^{\prime },\pi }^{+}\left(u,i\right)$ are the excitatory and inhibitory weights from neuron $\pi^{\prime }$ and π in the RNN for the client–service pair (u,i).

The term $q_{\pi }\left(u,i\right)$ is the probability that neuron π of the RNN (u,i) is excited, i.e., $0<q_{\pi }\left(u,i\right)<1$, and the expression (3) indicates that $q_{\pi }\left(u,i\right)$ can be computed as the ratio of excitatory (in the numerator) and inhibitory (in the denominator) signals that are entering neuron π. The largest among all the $q_{\pi }\left(u,i\right)$ for a given (u,i) represents the path that will be chosen at a given decision step as indicated in (10). The excitation between the different neurons is represented by the second term in the numerator of (3), while the “competition” between the paths that one may choose is represented by the inhibitory term (the third term) in the denominator. The numerator contains a positive term $\Lambda_{\pi }\left(u,i\right)$ which ensures that the probabilities are also positive.

The expression (4) implies that neurons are not self-excitatory or self-inhibitory, while (5) is the total firing rate $r_{\pi }\left(u,i\right)$ of neuron π in the RNN for the client–service pair (u,i).

We first initialized all the networks with probabilities $q_{\pi }\left(u,i\right)=0.5$ so that initially all the neurons are neutral with respect to the choice of path, by setting the weights to the following values:

(6) $$\begin{array}{ccc} W_{\pi ,\pi^{\prime }}^{+}\left(u,i\right) & = & W_{\pi ,\pi^{\prime }}^{-}\left(u,i\right)=w>0\;for\;\pi ,\pi^{\prime } \end{array}$$

(7) $$\begin{array}{ccc} r_{\pi }\left(u,i\right) & = & 2\left(N\left(u\right)-1\right)w,\;\lambda =\Lambda_{\pi }\left(u,i\right)=\lambda_{\pi }\left(u,i\right),\;\forall \;\pi ,u,i, \end{array}$$

So that:

(8) $$0.5=\frac{\lambda +0.5(N(u)-1)w}{\lambda +2.5(N(u)-1)w},\;or\;\lambda =1.5\left(N\left(u\right)-1\right)w,\;yielding\;q_{\pi }\left(u,i\right)=0.5,\;for\;any\;w>0.$$

#### The Reinforcement Learning Algorithm

The **RL algorithm**, which is shown below to be of complexity $O(n^{2})$ in the number of arithmetic operations executed after each service request is completed, where *n* is the total number of possible connections from clients to services, proceeds as follows:

1. After **server *f* is chosen to execute service *i* to satisfy the request of client *u***, the resulting total client response time, i.e., the first term in Equation (1), is measured from the simple difference of the time-stamp when the request is sent by the client and the time-stamp when the result is received by the client. The traffic rate on the path being used is also measured during the transfer of the request and the second term of (1) is obtained from a table look-up of power consumption versus traffic rate. As a result, the value of the goal function $G_{t}\left(u,i,f\right)$ is computed with two multiplications and one addition, from the measurement data regarding client response time and path consumption.
2. The historical value of the “reward”, defined as the inverse of the goal, i.e., ${\left[G\left(u,i,f\right)\right]}^{-1}$, defined as Θ(u,i,f) is updated:
  (9) $$\Theta_{t}\left(u,i,f\right)=\delta .\Theta_{t-1}\left(u,i,f\right)+\left(1-\delta \right)\frac{1}{G_{t}\left(u,i,f\right)},$$
  where 0<δ<1 is used to give more or less importance to the recent measurements. Note that this requires one division, two multiplications and one addition.
3. Subsequently, the RNN weights are updated:
  $$\begin{array}{ccc} & & do\;\forall \pi \in \Pi \left(u\right):\;r_{\pi }\left(u,i\right)\leftarrow \sum_{\pi^{\prime }\in \Pi \left(u\right)}\left[W_{\pi ,\pi^{\prime }}^{+}\left(u,l\right)+W_{\pi ,\pi^{\prime }}^{-}\left(u,l\right)\right]\;,\\ & & Requiring\;a\;total\;of\;2n\left(n-1\right)\;additions\;for\;n\;distinct\;paths\;i.e.,\;O\left(n^{2}\right)\\ & & If\;{\left[G_{t}\left(u,i,f\right)\right]}^{-1}\geq \Theta_{t-1}\left(u,i,f\right)\;do\;\forall \;\pi^{\prime }\in \Pi \left(u\right),\;\pi^{\prime }\neq \pi \left(u,i,f\right):\\ & & \left(a\right):\;W_{\pi^{\prime },\pi \left(u,i,f\right)}^{+}\left(u,i\right)\leftarrow W_{\pi^{\prime },\pi \left(u,i,f\right)}^{+}\left(u,i\right)+\frac{1}{G_{t}\left(u,i,f\right)},\\ & & Requiring\;(n-1)[multiplications+divisions+additions]\;i.e.,\;O(n),\\ & & \left(b\right)\forall \pi \neq \pi \left(u,i,f\right),\;\pi \neq \pi^{\prime }:\;W_{\pi^{\prime },\pi }^{-}\left(u,i\right)\leftarrow W_{\pi^{\prime },\pi }^{-}\left(u,i\right)+\frac{1}{\left(N\left(u\right)-1\right)G_{t}\left(u,i,f\right)},\\ & & Requiring\;\left(n-1\right)\left(n-2\right)\left[multiplications+divisions+additions\right]\;i.e.,\;O\left(n^{2}\right),\\ & & Else\;do\;\forall \;\pi^{\prime }\in \Pi \left(u\right),\;\pi^{\prime }\neq \pi \left(u,i,f\right):\\ & & \left(a^{\prime }\right)\;W_{\pi^{\prime },\pi \left(u,i,f\right)}^{-}\left(u,i\right)\leftarrow W_{\pi^{\prime },\pi \left(u,i,f\right)}^{-}\left(u,i\right)+\frac{1}{G_{t}\left(u,i,f\right)},\\ & & Requiring\;(n-1)[multiplications+divisions+additions]\;i.e.,\;O(n),\\ & & \left(b^{\prime }\right)\;\forall \pi \neq \pi \left(u,i,f\right),\;\pi \neq \pi^{\prime }\;:\;W_{\pi^{\prime },\pi }^{+}\left(u,i\right)\leftarrow W_{\pi^{\prime },\pi }^{+}\left(u,i\right)+\frac{1}{\left(N\left(u\right)-1\right)G_{t}\left(u,i,f\right)},\\ & & Requiring\;\left(n-1\right)\left(n-2\right)\left[multiplications+divisions+additions\right]\;i.e.,\;O\left(n^{2}\right). \end{array}$$
4. Then, to prevent the weights from constantly increasing:
  $$\begin{array}{ccc} & & do\;\;\forall \pi \neq \pi^{\prime }\;such\;that\;\pi ,\pi^{\prime }\in \Pi \left(u\right):\\ & & \left(c\right)\;W_{\pi ,\pi^{\prime }}^{+}\left(u,i\right)\leftarrow W_{\pi ,\pi^{\prime }}^{+}\left(u,i\right)\;\frac{r_{\pi }\left(u,i\right)}{\sum_{\pi^{\prime }\in \Pi \left(u\right)}\left[W_{\pi ,\pi^{\prime }}^{+}\left(u,i\right)+W_{\pi ,\pi^{\prime }}^{-}\left(u,i\right)\right]}\;,\\ & & \left(d\right)\;W_{\pi ,\pi^{\prime }}^{-}\left(u,i\right)\leftarrow W_{\pi ,\pi^{\prime }}^{-}\left(u,i\right)\;\frac{r_{\pi }\left(u,i\right)}{\sum_{\pi^{\prime }\in \Pi \left(u\right)}\left[W_{\pi ,\pi^{\prime }}^{+}\left(u,i\right)+W_{\pi ,\pi^{\prime }}^{-}\left(u,i\right)\right]}\;,\\ & & Requiring\;n\left(n-1\right)\left[multiplications+divisions\right]\;and\;2n\left(n-1\right)\;additions\;i.e.,\;O\left(n^{2}\right). \end{array}$$
5. Finally, with these updated values of the weights, we compute all the $q_{\pi }\left(u,i\right)$ using the system of Equation (3), which is a fixed point iteration of complexity $O(n^{2})$.
6. Then, we obtain the new value of the best choice of path for the request from client *u* for service *i*, including the path itself and the Fog server at the end of the path:
  (10) $$\pi^{*}\left(u,i,f^{*}\right)=\arg\max\left\{\;q_{\pi (u,i,f)}\left(u,i\right)\;:\;\pi \left(u,i,f\right)\in \Pi \left(u\right)\;\right\}\;\;,$$
  which uses (n−1) comparison operations if we use Bubblesort, or O(logn) if we use a more sophisticated sorting algorithm.

The goal function includes the end-to-end response time (including round-trip network delay and the service time needed to satisfy the request), plus the path power consumption. Path power consumption is the sum of the power consumption on each node in the path, and the node power consumption is computed from the value of the traffic that a request generates. The end-to-end response time is measured constantly each time a specific source-destination path is used, since it includes both the network round-trip delay and the destination server’s service time for the client. When a decision has to be taken, the source is known and the decision is then to select both the destination (where the service is resident) and the path which minimizes the goal function.

The algorithm that we described is run on the SDN controller to find the path $\pi^{*}\left(u,i,f^{*}\right)$ and determines the Fog node $f^{*}$ in (10), so that the service request of the client *u* for service *i* can be satisfied.

## 3. Linking Network Power Consumption to SDN Switch Traffic Rate

**Figure 2:**
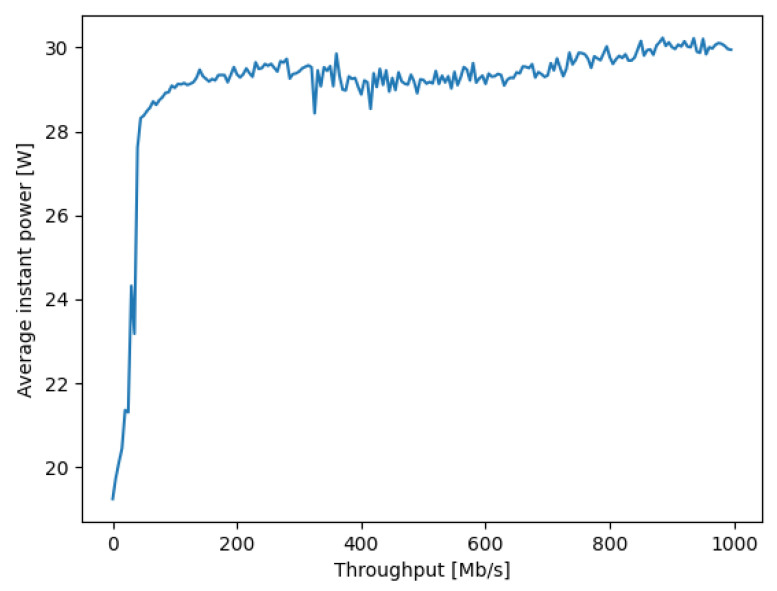
The dependence of the instantaneous power consumption on the traffic load of an Intel NUC that is used as a SDN switch or router. The *y* axis is the power consumption in Watts, averaged over 30 distinct measurements, against the traffic values provided in the *x* axis in Mb/s.

The total energy consumption E(π(u,i,f)) for the network connection of client *u* from service *i* at Fog node *f*, using the network path π(u,f) in the expression (1) is estimated as the sum of the energy consumption of each network switch (i.e., SDN router) *x* on path π(u,f), which transfers a total of D(u,i) packets in either direction when the request from *u* is made and satisfied by *i* at Fog node *f*:

(11) $$E\left(\pi \left(u,i,f\right)\right)=\sum_{x\in \pi (u,i,f)}D\left(u,i\right).\frac{P_{x}\left(\tau_{x}\right)}{\tau_{x}},$$

where $\tau_{x}\geq 0$ is the total traffic rate in Mb/s that is being carried by node *x*, and $P_{x}\left(\tau_{x}\right)$ is the power (in watts) consumed at the network switch *x* when it carries a traffic rate $\tau_{x}$. An example of $P_{x}\left(\tau_{x}\right)$ versus $\tau_{x}$ is shown in Figure 2. Note that in the expression for the energy consumption (11), the term:

(12) $$\frac{P_{x}\left(\tau_{x}\right)}{\tau_{x}},\;\tau_{x}\in \left[0,+\infty \right],$$

is given in Joules/Mb due to the fact that power is measured in Watts, or Joules/second, while the traffic rate $\tau_{x}$ is measured in Mb/s, so that the expression (12) is in Joules/Mb. Since D(u,i) is the total amount of data in Mb transferred back and forth, the expression (11) is in Joules.

**Figure 3:**
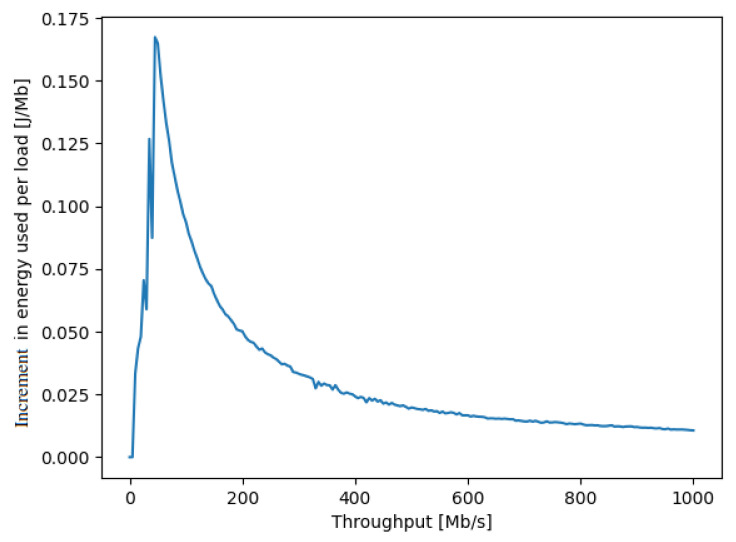
The increment in the amount of energy in *Joules per Mb* transported through a NUC acting as a SDN switch (shown on the *y* axis), as a function of the ongoing traffic rate in Mb/s passing through the NUC (shown on the *x* axis). This curve shows that if we operate the NUC at the left-hand side of the peak of the curve, increasing the traffic will also increase the energy per unit traffic, while if the NUC is operated at the right-hand side of the peak, then as we add on more traffic through the NUC, the energy per Mb actually decreases.

Another metric of interest is the energy consumption increment (ECI) of the SDN switch *x*, measured in Joules per Mb, which indicates the additional energy consumption per unit of data transferred. The ECI can be obtained directly from $P_{x}\left(\tau_{x}\right)$ by calculating its derivative:

(13) $$ECI_{x}\left(\tau_{x}\right)=\frac{dP_{x}\left(\tau_{x}\right)}{d\tau_{x}},\;\tau_{x}\in \left[0,+\infty \right],$$

and a measured experimental example is shown in Figure 3 in Watts per Mb/s, or equivalently in Joules per Mb.

### Measuring the Power Characteristic of the SDN Switches

The SDN switches (or forwarders) used in our experiments are Intel Next Unit Computers (NUC) [45] that run Open vSwitch [25]. Although in this paper we only focus on these devices, the approach we take can be applied to any other relevant network hardware.

The measurement of the instantaneous power drawn by a NUC during data transfer was performed in the experimental setup shown in Figure 1. For each individual power measurement, a fixed level of traffic in Mb/s was supplied by another NUC, to the NUC being measured acting as a SDN switch, and the experiment was carried out for successively increasing traffic levels, as shown in Figure 2.

The electronic circuit which is used to condition the signal obtained from a sensor which measures the current is based on precision operational amplifiers. The Hall effect-based current sensor ACS712-05 (0–5 Ampères current range) is galvanically isolated from the copper conduction path, integrated into the IC, which is used to pass the measured current. This path was connected in series with the supply wire on the constant DC voltage side at $U_{DC}=19.5$ V, of the AC adapter used for the NUC’s as shown in Figure 1. The output signal from the sensor is amplified in a single-ended amplifier and then converted to the differential form. The instantaneous value of the measured power can then be found from the following relationship:

(14) $$P=U_{DC}.i=U_{DC}\frac{U_{m}}{k_{u}S}=AU_{m},\;in\;Watts\;,$$

where S=185 mV/A is the sensitivity of the current sensor, and $A=U_{DC}/\left(k_{u}S\right)=52.7$ A is a constant with $k_{u}=2$, which is related to the instrumentation, and $U_{m}$ is the measured output voltage of the single-sided differential converter shown at “channel 1” of Figure 1, which results from the Hall-effect measurement of the NUC input current.

In order to register the waveforms of the instantaneous power, a professional multi-channel 16-bit resolution measurement card, installed on a PC computer, was used with 1 Khz sampling frequency. To synchronize the measurements, a photodetector was connected to another channel of the measurement card in order to detect a change in the brightness of the screen in the monitor connected to the transmitting NUC. Such a simple solution enabled the generation of a marker signaling the start of a test procedure, providing galvanic isolation at the same time. To reduce the effect of noise and interference, thirty separate measurements were repeated for the power consumption as a function of incoming and outgoing traffic, and the results are summarized in Figure 2. Then, we extracted the difference of the energy consumption between the basic level for zero traffic and the value for a given traffic level, and the increase in energy consumption per traffic volume in Mb is presented in Figure 3.

## 4. The Test-Bed and the Experimental Results

**Figure 4:**
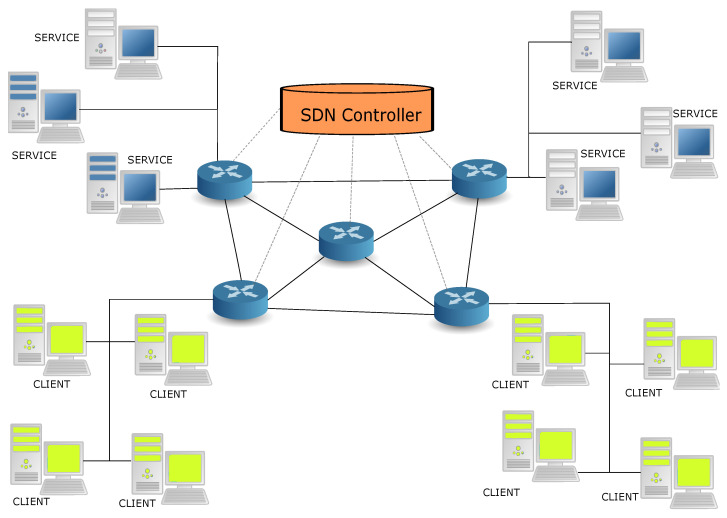
The architecture that we used for the experiments is shown, including the clients and services (resident on servers), plus the SDN network with a SDN controller and 5 SDN switches (the round blue objects) with 8 links between the switches. All the SDN switches are implemented on Intel NUCs. Two switches support the connections to services, while two other switches support the connections to the clients.

The test-bed that is used for our experiments is shown in Figure 4. It is a network of five SDN switches plus a SDN controller shown in red. All five SDN switches are installed in NUC [45] machines running OpenVSwitch version 2.12.0, on of Linux Kali version 2020.2, kernel version SMP Debian 5.5.17-1kali1 (21 April 2020). The SDN controller is a separate NUC machine which only runs the ONOS SDN controller software utilizing the Open-Flow Protocol version 13+ [57]. Prior work on similar test-bed organizations can be found in [17].

In Figure 4, two of the NUC machines, FD2,FD5, also act as Fog servers, each of which supports all the services. Two other NUC machines, FD3,FD4, support the clients. All NUC machines also act as SDN switches, including FD1, which is not used as a host for services or clients. This topology has been selected to easily allow for our experiments. Any client *u* in Figure 4 can connect to any service instance in the system. From the client’s point of view, it does not matter *where* the service is deployed. Note that for any client–service pair, there are multiple paths possible in the 5-node SDN switch topology.

At the client level, each service *i* is designated with a specific and unique *dummy* IP address. When a client requests a specific service, the SDN controller designates on the fly the *current optimal server* using the RL algorithm detailed in Section 2.1, and it also uses the open-flow protocol to assign the appropriate path to the SDN switches that are concerned by this decision. Details on the use of a SDN to implement both the network routing and the software support for allocating service requests to specific service instances can be found in our earlier conference paper [44].

In order to test the performance of the RL-based algorithm, we developed a set of experiments where the instants at which the users or clients make requests, as well as their selection of the service they request, is randomized, so as to decouple the behavior of the workload from the control algorithm itself. Thus, each client $C_{u}$ sends a request to a randomly chosen service $S_{1}$ or $S_{2}$ at a random instant $T_{u,i}$ which is uniformly distributed in the time window $\left[\;T_{0},\;T_{0}+T_{max}\;\right]$ where $T_{max}=10$ s.

We distinguish between Type 1 clients which have a preference for service $S_{1}$, and the probability of choosing this service which is $P_{1}\left(S_{1}\right)=0.75$, while Type 1 clients choose $S_{2}$ with probability $P_{1}\left(S_{2}\right)=0.25$. Type 2 clients prefer the second service with probability $P_{2}\left(S_{2}\right)=0.75$ and $P_{1}\left(S_{1}\right)=0.25$. Recall that each instance of $S_{1}$ and $S_{2}$ is installed on all servers, and that each server is able to process multiple requests simultaneously.

### 4.1. Experimental Results Regarding the Response Time Only

**Figure 5:**
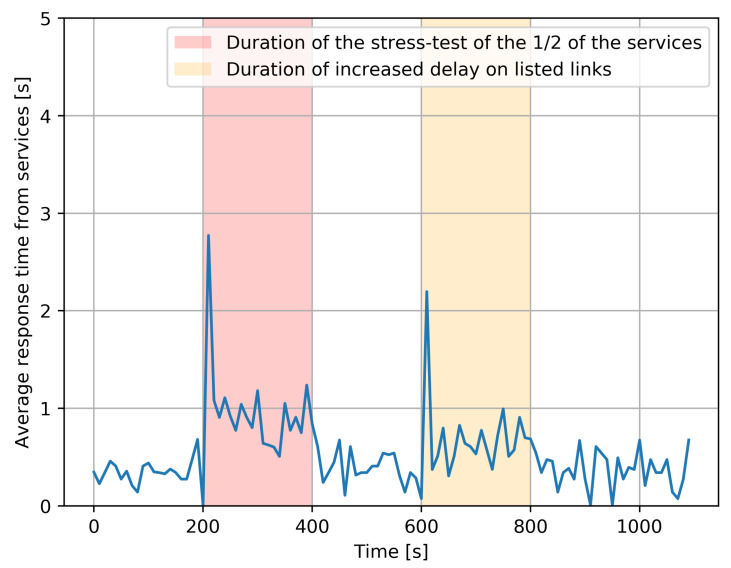
The average response time experienced by all clients for the services, measured over some 1000 s, averaged over five distinct experiments with identical parameters.

**Figure 6:**
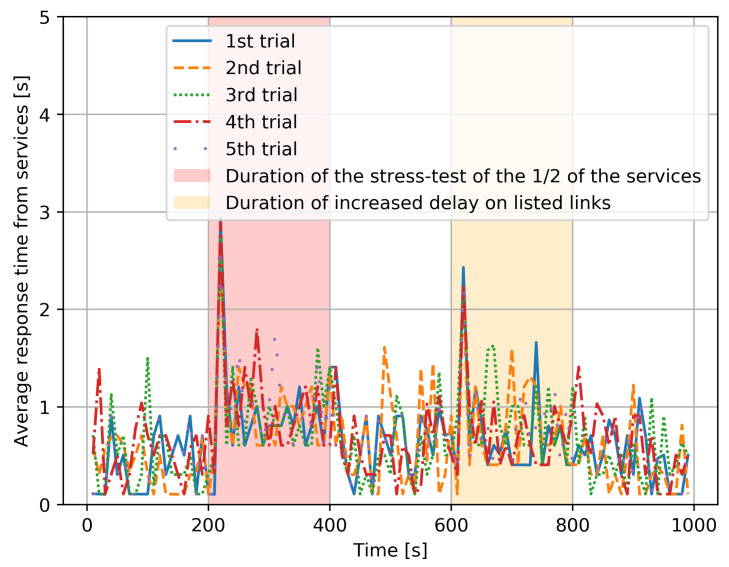
Individual variations in the average response time observed for each of the five distinct experiments with identical parameters.

The following experiment was run to evaluate the effect of the RL-based control algorithm of Section 2.1 with a simplified goal function (1) where we have set α=1, i.e., where energy consumption in the network is *not* taken into consideration:

1. In the time window $\left(T_{0},T_{0}+T_{max}\right)$, each client $C_{u}$ sent one request to one service, and all services are available and are responding as fast as they can. The resulting response time for the m−th request which is satisfied for each user is denoted $T_{m},\;m\geq 1$, from the instant when the user makes the request to the instant when the response is received back at the use. The overall average value of the *n* requests that are satisfied over the duration of the experiment is denoted:
  (15) $$T=\sum_{m=1}^{n}T_{m}.$$
  This experiment was repeated five times consecutively, and the overall average response time, which is an “average of averages”, taking the average of the 5 values of the individual average values *T*, is shown in Figure 5, in the part of the figure which does not have a background color.
2. Secondly, the same experiment was run with a “stress test”, which is an additional program that executes 50% of the services which have been chosen at random, and simultaneously increases the CPU utilization rate to 100%, resulting in a substantial increase in the time required to process the corresponding service requests. Its effect is shown with a red background in Figure 5. Interestingly, we notice that the effect of the stress test is mostly seen at the beginning of the “red period”, due to the RL-based adaptive control which dynamically shifts the load towards those service instances which do not have an overload. However, since 50% of services are systematically affected, we do have an increase in average response time. After the stress test ends, everything goes back to the prior condition.
3. Thirdly, during the time span shown with an orange background in Figure 5, the links between $\left(FD_{2},FD_{3}\right)$, $\left(FD_{4},FD_{5}\right)$ experience a major increase in delay caused by a DDoS attack. As a result, the clients also experience a major increase in response time due to the additional transfer delay of requests and of the corresponding responses. Again, we observe that the worse effect is at the beginning of the “yellow period”, since the RL-based adaptive control shifts the workload to the longer two-hop paths that are not under attack. Obviously, an increase in response time still occurs because of the longer paths, but it is not as bad as that at the beginning of the attack since the RL-based control has been able to avoid the links which are under attack. After the DDoS attack ends, the average response times fall back to “normal”.
4. The same results are shown in Figure 6 for each of the five distinct experiments, and we see that each distinct experiment has a behavior that is very similar to the average behavior shown in Figure 5.

### 4.2. Experiments Concerning Energy Optimization

**Figure 7:**
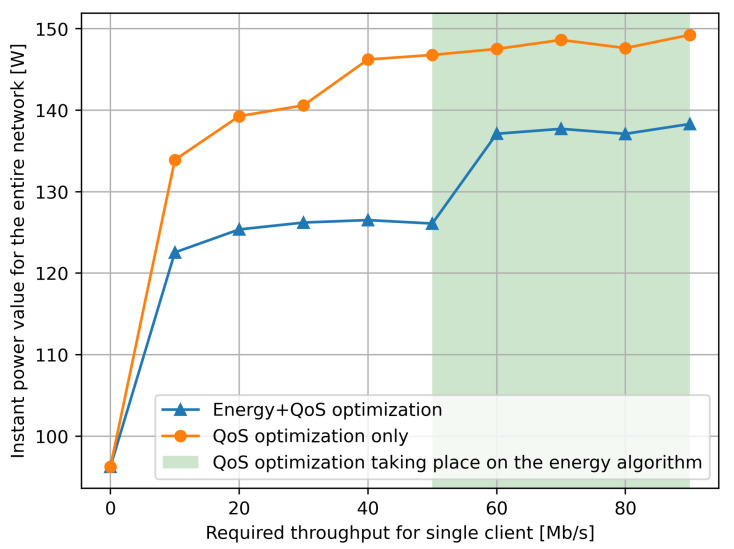
Instantaneous power consumption in the SDN switch part of the network, measured as a function of the clients’ data throughput towards the servers which are supporting the services. The power value is deduced from traffic measurements and from the power versus traffic data in Figure 2. We see that when the RL controller takes power into account, a power savings of the order of 15% occurs.

In the next set of experiments that we describe in this section, we used the full expression in the goal function (1) with α=0.3 so that the energy component of the goal function is 70% of the total composite value. As shown in Figure 7, in different experiments we vary the size of the data transferred by the client $C_{u}$ to its corresponding service request $S_{i}$, so that we also vary the corresponding power consumption of the network according to the characteristic shown in Figure 2 for each individual NUC-based SDN switch that is traversed on each path.

We simplify the experiments by letting each client have exactly the same transfer rate $\tau_{r}$ in Mb/s from client to server, whose value is shown on the *x* axis of Figure 7. Note that the power consumption at *each NUC used as a SDN switch* can then be estimated from the value of $\tau_{r}$ using the results in Figure 2.

**Figure 8:**
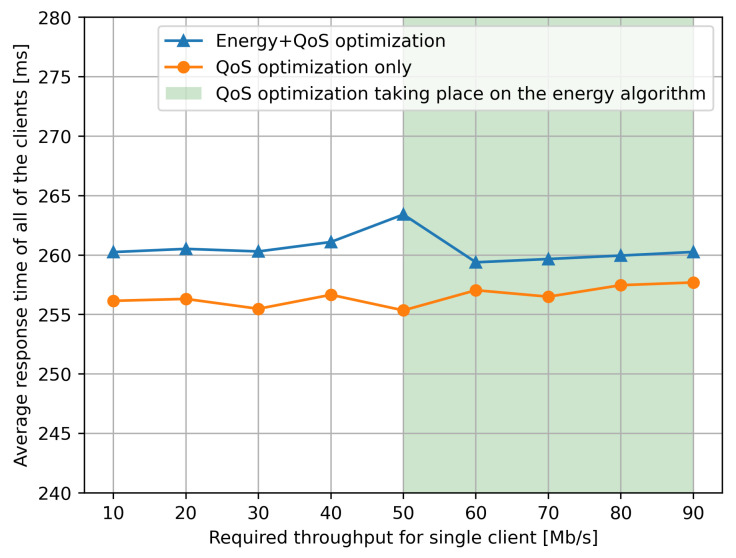
We observe the very significant stability of the average response time of the system over a long period of 500 s, when either QoS optimization is, or QoS and energy optimization are conducted by the RL-based control algorithm. This illustrates the ability of the RL control to react to changes in load represented by ongoing requests by the clients to the services, maintaining the average response time at a low level. We see that the reduction in power consumption observed in Figure 7 comes at an increase of less than 2% (5 parts in 250) in the average response time.

For each value of throughput per client–server pair, and for each value of $\tau_{r}$, we repeat the following experiment 10 times, so that the results we report are an average over the 10 experiments for each $\tau_{r}$. Each individual experiment is run as follows using the RL control with the goal function (1) and α=0.3, as well as (separately) by setting α=1, i.e., without energy optimization:

1. Each client $C_{u}$ sends a request with a stream of data which is prepared so that its throughput is $\tau_{r}$, and $\tau_{r}$ is varied between 10 and 90 Mb/s, as shown by the *x* axis of Figure 7 and Figure 8.
2. Each client sends one request in the 10 s time window as described in the previous section.
3. The actual throughput is measured at the SDN switches (NUCs) and the instantaneous power consumption is computed from Figure 2.

The results we observe in Figure 7, regarding the total average power consumption (over the ten experiments), indicate that if we just try to minimize the response time only with α=1 (the red line), the obvious result is that total power consumption increases substantially as compared to the case where we have α=0.3, and both power and response time are minimized (the blue line). However, what we point out in the “white area” of Figure 7 for the “blue line” is that the response time in the goal has little effect for smaller values of $\tau_{r}$, while when we increase $\tau_{r}$ *above* 50 Mb/s, the response time starts having a significant effect on the goal function and therefore it forces the RL-based control to use more power by selecting longer paths.

Note that the green selection on the Figure 7 only refers to the energy optimization. This green selection shows where the QoS part of the optimization forced the energy optimization to use another forwarder. We can observe it in this rapid jump of the measured power usage.

The data from Figure 7 are supported by the average response time shown in Figure 8 over a long time period of 500 s under exactly the same conditions. We see that the average response time is obviously higher if the optimization addresses both the response time and power consumption (blue curve).

However, we also see (counter-intuitively) that the average response time is actually lower (blue line) when $\tau_{r}>50$ Mb/s as compared to $\tau_{r}=50$ Mb/s, simply because the response time portion of the goal used by the RL algorithm “kicks in” strongly when the response time degrades, by selecting longer but less loaded paths in the network.

**Figure 9:**
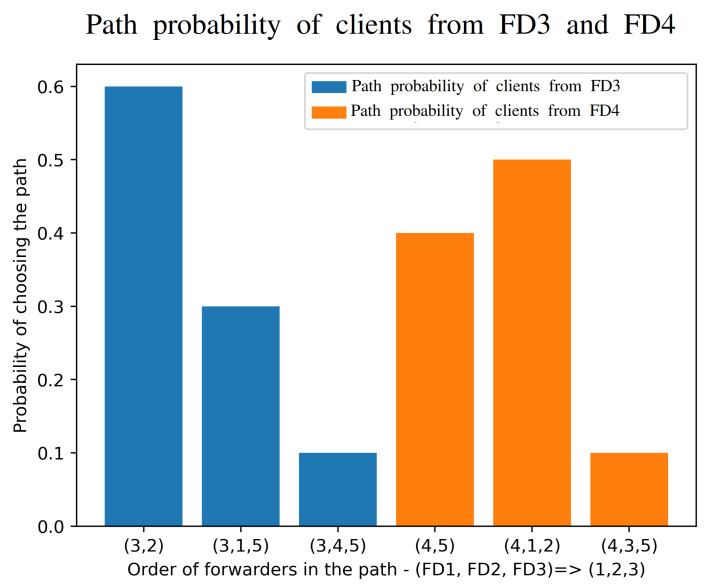
Measured relative frequency with which the optimization algorithm chooses the different network paths in Figure 4.

The variability of the paths used is shown in our measurements of Figure 9 which clearly indicate that different clients will be adaptively using different paths (of different lengths) in order to effect a dynamic online compromise between response time and power consumption.

## 5. Conclusions

In this paper, we extended our previous work on the dynamic QoS optimization of a Fog network [44] in the presence of randomly changing workloads of users or clients requesting services when a SDN is used to control the system.

The extension was to introduce the important issue of energy consumption. Combining power with QoS, we detailed the manner in which reinforcement learning can be used, together with random neural networks, to dynamically control the client-to-service allocation process so as to minimize a composite goal function combining QoS and energy.

In order to address this issue, we needed to acquire accurate load dependent data on the power consumption of SDN switches (or routers). Thus, as a first step, we measured the power consumption of SDN switches to the traffic rate they carry, and reported the accurate power consumption characteristics of the routers which were not previously available in the literature.

Using these results in a network-based interconnected test-bed of clients, servers, SDN switches and a SDN controller, we reported the performance of the resulting system both in terms of response time and power consumption. The results have shown how the system adapts to carry out a trade-off between response and power optimization, as well as the stability of the control scheme in the presence of long random sequences of service requests over hundreds of seconds. Our experiments also reveal that, with the specific goal function that we used that include power consumption, the power consumption of the system during the experiments was reduced by approximately 15% with respect to the case where only QoS was being optimized, while the average response time for the clients only increased by 2%. This indicates that power savings can be made through control policies such as ours, with little negative impact on QoS.

While this work addressed the combination of response time in the servers and the network, and the power consumed in the network, it also poses the question of the electrical power consumed in the servers. Thus, our future work will investigate a further level of optimization, combining both network and server energy, together with network and server QoS. Combining this with security issues will also be a further development of this work.
